# Optimization of the Fermentation and Preparation of the Wettable Powder Formulation of *Bacillus velezensis* F0b

**DOI:** 10.3390/microorganisms13030560

**Published:** 2025-03-01

**Authors:** Jiaqi Wen, Nana Pi, Fengting He, Yuhao Zeng, Qunfang Weng, Jianjun Luo

**Affiliations:** 1 College of Plant Protection, South China Agricultural University, Guangzhou 510642, China; wjiaqi168@163.com (J.W.); 15519917290@163.com (N.P.); heft0101@163.com (F.H.); 13533061123@163.com (Y.Z.); 2 Key Laboratory of Bio-Pesticide Innovation and Application of Guangdong Province, Guangzhou 510642, China

**Keywords:** *Bacillus velezensis*, formulation, biopesticides, wettable powder

## Abstract

*Bacillus velezensis* is an effective biocontrol bacterium, with its microbial pesticides showing promise in biological control. In this study, we optimized the medium and conditions for fermenting strain F0b, developed a wettable powder formulation, and assessed its efficacy against *Botrytis cinerea*. We screened carriers, wetting agents, dispersants, and UV protectants compatible with F0b, determining the optimal ratio and dosage. The best medium for F0b fermentation included rice flour (3.472%), ammonium chloride (4.898%), and disodium phosphate (1.871%). The ideal fermentation conditions were a 20% inoculum volume, 40 °C temperature, 80% sterile water, and a 72 h fermentation time, yielding a viable count of 1.33 × 10^10^ CFU/mL. The final formulation contained 54.7% *Bacillus velezensis* dried powder, 27.3% kaolinite carrier, 16% wetting agent (3:7 ratio of sodium dodecyl sulfate to sodium lignin sulfonate), and 2% ascorbic acid as a UV protectant. All quality indicators met national standards, with a viable bacteria concentration of 7 billion CFU/g. Field trials showed that the F0b wettable powder effectively controlled *Botrytis cinerea*, with a disease index significantly lower than the control group. Control efficacy ranged from 50.58% to 73.14% over 7 to 14 days, demonstrating the commercial potential of this formulation.

## 1. Introduction

*Bacillus velezensis* is primarily isolated from plant tissues and rhizosphere soil. It exhibits strong inhibitory effects on various plant pathogens. Recent studies have indicated that it possesses both antibacterial and antifungal properties, making it effective in controlling pathogenic fungi [1,2,3] and bacteria [4,5]. *B. velezensis* can produce a wide range of bioactive compounds, including antibiotics, lipopeptides, and bacteriocins, which have antifungal and antibacterial properties [6,7,8]. These compounds inhibit the growth of phytopathogens by disrupting their cell membranes or interfering with their metabolic processes. Additionally, it can induce systemic resistance in plants, enhancing their defense mechanisms against a broad range of diseases. It enhances the expression of auxin and ethylene (ET) hormone signaling-related genes, as well as defense genes associated with multiple signaling pathways. This activation induces plant resistance, leading to improved disease resistance and growth promotion [9,10]. The application of *B. velezensis* in biopesticides is becoming increasingly prominent, highlighting its considerable potential in managing plant fungal and bacterial diseases effectively [11,12,13].

Since *Bacillus* spp. can form endospores that withstand heat and desiccation, they are well-suited for formulation into stable dry powders with extended shelf lives, making them an ideal choice for microbial pesticide development [7,14]. Currently, formulations of *Bacillus* spp. primarily mainly include dry flowable formulations [15], wettable powders [15], suspensions, water-dispersible granules, and emulsifiable concentrates [16]. In recent years, *B. velezensis* has been developed and applied on a large scale, yet much of the formulation research remains in the experimental phase, with limited studies available. Only a few studies have explored formulations such as soluble granules, wettable powders, and water-dispersible granules. Currently, the formulation products that have been successfully commercialized include Serenade^®^ ASO (containing *B. velezensis* QST 713), developed by Bayer, and water-dispersible granules (containing *B. velezensis* CGMCC No. 14384), produced by Sichuan Baishi Dongwang Biotechnology Co., Ltd., Chengdu, China. However, research on the development process, stability optimization, and industrial production of *B. velezensis* formulation products remains limited, which hinders their broader adoption and application. Therefore, conducting in-depth studies on the formulation design, production process optimization, and application efficacy of *B. velezensis* in diverse environments holds substantial theoretical significance and practical value. Such research can provide a scientific foundation and technical support for the industrial development of this strain.

Currently, wettable powder remains a preferred option for processing microorganisms into pesticide formulations. The formulation, composed of live microorganisms mixed with wetting agents, dispersants, and adjuvants in specific proportions, is ground to a fine particle size and designed to be wetted and suspended in water. It offers advantages such as fine particle size, easy wetting, high active ingredient content, low cost, broad applicability, and ease of transport [17]. Considering the previously mentioned characteristics of *B. velezensis*, which make it suitable for formulation into a stable dry powder with an extended shelf life, wettable powder emerges as an ideal formulation choice.

Strawberry (*Fragaria × ananassa* Duch.) is an important economic crop in China. As a fresh fruit, strawberries are characterized by high water content, softness, and susceptibility to mechanical damage. In addition, they are highly sensitive to fungal infections, making them easy to rapidly rot. Strawberry gray mold caused by *Botrytis cinerea* is one of the most common and destructive diseases affecting strawberries [18]. This pathogen can cause infections throughout the growth cycle, including flowering, fruit ripening, and even harvesting and subsequent storage and sale [19]. While the application of fungicides is the most common and effective method to control *Botrytis cinerea*, prolonged use leads to the development of resistance in the pathogen [18,20,21,22]. Therefore, it is very necessary to develop alternative biological control methods, such as the use of fungi and bacteria for the prevention and control of gray mold. Yang et al. [8] demonstrated that the cell-free supernatant (CFS) from *B. velezensis* YTQ3 effectively inhibited the hyphal growth and spore germination of *Botrytis cinerea*, thereby suppressing its growth. Therefore, we believe that *B. velezensis* may exhibit strong activity against *Botrytis cinerea* and holds promise as a potential biological control agent for this pathogen.

This study developed a formulation for *B. velezensis* F0b wettable powder and evaluated its efficacy in controlling strawberry gray mold, providing a scientific foundation for the development and application of *B. velezensis* F0b as a novel microbial pesticide.

## 2. Materials and Methods

### 2.1. Bacterial Strain and Culture Conditions

*B. velezensis* strain F0b was originally isolated from tomato leaves collected from a field in Guangzhou, Guangdong, China, and identified by morphological and physiological experiments and ITS gene analysis.

*B. velezensis* F0b was inoculated on Nutrient Agar (NA) medium (beef extract 3 g L^−1^, peptone 10 g L^−1^, yeast powder 1 g L^−1^, agar 18 g L^−1^, glucose 10 g L^−1^) and cultured at 28 °C for 24 h. The activated F0b was inoculated into Luria–Bertani (LB) liquid medium (yeast powder 5 g L^−1^, tryptone 10 g L^−1^, NaCl 10 g L^−1^) for 24 h to obtain the liquid culture. The liquid culture of F0b was inoculated into LB liquid medium at a 1% inoculum amount for 16 h to obtain the fermentation liquid. In total, 10% of *B. velezensis* F0b fermentation liquid was added to the fermentation medium, and the culture was incubated at 35 °C with shaking at 180 r/min for 72 h to obtain the fermentation product. The number of viable bacteria in the fermentation product was determined using the plate count method.

### 2.2. Identification of Strain F0b by 16S rDNA Sequence Analysis

#### 2.2.1. Identification by Morphological Experiments

The preserved F0b strain was streaked onto NA medium and incubated at 28 °C for 24 to 48 h. After the growth of isolated colonies, the colony morphology was observed, including characteristics such as size, shape, odor, color of the colony and surrounding culture medium, surface texture, edge appearance, transparency, glossiness, and consistency. Photographs were taken to document the observations.

#### 2.2.2. Identification by 16S rDNA Sequence Analysis

In this experiment, strain F0b was extracted using the TSINGKE plant DNA extraction kit (Qingke Biotechnology Co., Ltd., Beijing, China), and DNA was extracted from the strain sample to obtain genomic DNA. The genomic DNA was used as a template, and the bacterial 16S rDNA universal primers 27F (5′-AGTTTGATCMTGGCTCAG-3′) and 1492 (5′-GGTTACCTTGTTACGACTT-3′) were used to amplify and sequence the target strain 16S rDNA. The components of the PCR reaction system are as follows: 45 µL of 1× TSE101 Mix, 2 µL of upper and lower primers, and 1 µL of DNA template. The reaction conditions were as follows: pre-denaturation at 98 °C for 2 min; amplification cycle at 98 °C for 10 s, 56 °C for 10 s, and 72 °C for 10 s for 35 cycles; final extension at 72 °C for 5 min.

After the PCR product was detected by agarose gel electrophoresis, the PCR amplification sample was sent to Beijing Qingke Biotechnology Co., Ltd. (Beijing, China) for sequencing analysis. The sequencing results were used to perform BLAST (https://blast.ncbi.nlm.nih.gov/Blast.cgi, access date 15 October 2023) comparison in the NCBI database to preliminarily identify the sample, and the phylogenetic tree was constructed using MEGA7.0 software.

### 2.3. Reagents

The ingredients used in the formulation, including carriers, dispersants, wetting agents, and UV protectants, were primarily sourced from Yuanye Biotechnology Co., Ltd., Shanghai, China.

Carriers included talcum powder (A1), diatomaceous earth (A2), kaolinite (A3), attapulgite (A4), bentonite (A5), and corn starch (A6). Wetting agents consisted of sodium dodecyl sulfate (B1), Morwet-EFW (B2), sodium dodecylbenzene sulfonate (B3), and sodium C14-C16 olefin sulfonate (B4). The dispersants used were NNO (C1), GY-D800 (C2), Prowet WG20 (C3), sodium lignin sulfonate (C4), and MF (C5). UV protectants included soluble starch (D1), ascorbic acid (D2), xanthan gum (D3), and sodium alginate (D4).

### 2.4. Optimizing Fermentation Medium Compositions for Bacillus velezensis F0b

#### 2.4.1. Fermentation Medium Screening by a Single-Factor Test

The initial fermentation medium was prepared using a mixture of soybean meal and peanut bran in a 6:4 ratio as the fermentation matrix. All matrices were crushed and passed through a 200-mesh sieve for later use. A total of 20 g of the fermentation matrix was weighed into a 250 mL conical flask, 4 g of water was added, and the mixture was sterilized at 121 °C for 30 min to obtain the initial fermentation medium. In subsequent experiments, different carbon sources, nitrogen sources, and inorganic salts were incorporated into this base medium.

Most of the chemical substances, including carbon sources, nitrogen sources, and inorganic salts, were purchased from Zhiyuan Chemical Reagent Company (Tianjin, China), while rice flour and corn flour were purchased from local supermarkets.

Screening was performed to identify suitable carbon sources, nitrogen sources, inorganic salts, and their optimal dosages. The carbon sources included rice flour, ethylene glycol, glucose, sucrose, soluble starch, corn flour, and yeast powder, with 5% added to the fermentation medium. Nitrogen sources, including ammonium chloride, sodium nitrate, soybean powder, tryptone, peptone, ammonium sulfate, millet powder, and yeast extract were tested, with 5%. Inorganic salts, including disodium phosphate, iron sulfate, zinc sulfate, copper sulfate, magnesium sulfate, manganese sulfate, potassium dihydrogen phosphate, and sodium chloride, were selected for the fermentation culture group, with 1%. Each treatment was repeated four times. The number of viable bacteria in the fermentation product of *B. velezensis* F0b was determined by the method described in Section 2.1.

The concentrations of carbon source, nitrogen source, and inorganic salt in the culture medium were screened at different levels, including carbon source and nitrogen source from 1 to 11% and inorganic salt from 1 to 7%.

#### 2.4.2. Fermentation Medium Optimizing by Response Surface Methodology 

The orthogonal test was conducted under the range of conditions obtained by the single-factor test. These medium compositions were further optimized for fermentation of *B. velezensis* F0b using response surface methodology based on the Box–Behnken design (Design Expert 13.0, Stat-Ease Inc., Minneapolis, MN, USA). As shown in the Table 1, three factors included were designed, each factor had 3 levels, and the number of viable bacteria was used as the response value.

### 2.5. Optimization of the Fermentation Conditions of Bacillus velezensis F0b

Factors of the fermentation condition for *B. velezensis* F0b production were, respectively, evaluated at various levels, including inoculum volume from 10 to 60%, culturing temperature from 20 to 50 °C, culturing time from 12 to 96 h, and water content (the ratio of sterile water to the total amount of both sterile water and fermentation materials) from 50 to 87.5%.

### 2.6. Preparation of Wettable Powder Formulation

First, a suitable carrier is selected to make the original powder. Next, screening experiments are conducted on wetting agents, dispersants, and UV protectants individually.

#### 2.6.1. Screening of Carriers

Six types of carriers (A1–A6) were selected and mixed with the fermentation of *B. velezensis* F0b and dried by air blasting to obtain the original powder. The spore survival rate, suspension rate, and wetting time in each formulation was determined to obtain the optimum carrier. The original powder containing the best carrier was used for subsequent experiments.

#### 2.6.2. Screening of Dispersant and Wetting Agent as Well as Determination of Their Amounts

Four wetting agents (B1–B4) and five dispersants (C1–C5) were added separately to *B. velezensis* F0b powder at 10%, with an untreated sample serving as the control. The indicators of each formulation were measured to identify the most suitable dispersant and wetting agent.

The selected wetting agent and dispersant were then mixed in various mass ratios (1:9, 2:8, 3:7, 4:6, 5:5, 6:4, 7:3, 8:2, 9:1), added to the powder at a total of 10%, and evaluated based on the same indicators to determine the optimal ratio.

Finally, the best compound additive, in concentrations ranging from 2% to 20%, was mixed with the powder, and the optimal concentration was selected.

The evaluation indicators for all formulations included the number of viable bacteria, wetting time, and suspension stability.

#### 2.6.3. Screening of UV Protectant

Based on the original powder and additives selected in Section 2.6.1 and Section 2.6.2, a preparation of *B. velezensis* was made. Four UV protectants (D1–D4) were added to the preparation at 2%. They were placed 40 cm away from the UV light (254 nm), exposed for 12 h and 24 h. After being diluted, they were coated on the plate. The optimum UV protectant was selected by measuring the bacterial viability. The preparation without protection served as the control.

### 2.7. Quality Index of the Formulation

#### 2.7.1. Bacterial Viability

The colony-forming units (CFUs) of the wettable powder formulation were determined using the dilution plate method. A total of 1 g of wettable powder was added to 10 mL of sterile water in a 50 mL conical flask, then shaken at 120 rpm for 1 h. Following this, a series of 10-fold gradient dilutions was performed. Two adjacent appropriate dilutions were selected, and 100 µL from each was spread onto NA medium plate to calculate the viable bacterial count.

#### 2.7.2. Suspensibility

A 2 g sample was added to 250 mL of standard hard water in a 250 mL measuring cylinder. The cylinder was then inverted 30 times within 1 min. Afterward, 225 mL of the sample was withdrawn, and the remaining 25 mL of the suspension at the bottom of the cylinder was dried in an oven at 105 °C until reaching a constant weight.

The suspension rate (*X*) was calculated according to the following formula:

$$X=10/9\times \frac{w-w1}{w}\times 100\%$$

where w is the quality of the sample (g) and w1 is the quality of the 1/10 residue (g).

#### 2.7.3. Wettability

The procedure for determining wetting time involves pouring 100 mL of standard hard water into a 250 mL beaker. A 5 g sample of the powder is then carefully and evenly added to the water, ensuring that the surface of the liquid is not disturbed. The time taken for the sample to completely wet is recorded to the nearest second. This experiment is repeated four times, and the results are averaged to obtain the final wetting time.

### 2.8. Field Experiments

The field experiment was conducted in Guangzhou, Guangdong Province, to evaluate the effect of the preparation on the gray mold of strawberries. Three concentrations of F0b WP were set, and the control agent 200 million CFU/g Trichoderma WP produced by Wanlihua Biotechnology Co., Ltd., Shanghai, China, was purchased from the market. The dosage of the agent is shown in the accompanying table. The test site consisted of five treatments, each with four replications, resulting in a total of 20 plots, each measuring 15 m^2^ and arranged in randomized blocks. The treatments were as follows: (1) 7 billion CFU/g F0b WP, 100 g/667 m^2^; (2) 7 billion CFU/g F0b WP, 200 g/667 m^2^; (3) 7 billion CFU/g F0b WP, 300 g/667 m^2^; (4) 200 million CFU/g *Trichoderma* WP, 200 g/667 m^2^; and (5) a control. The pesticide was applied by spraying the prepared solution onto the strawberry fruit until the leaf surface was evenly moistened, with slight dripping of the liquid. Two applications were carried out, with a 7-day interval between sprays.

Sampling was performed at five points in each plot, with 50 fruits surveyed at each point. The total number of fruits and the number of diseased fruits were recorded, and the disease incidence rate and control efficacy were calculated 7 to 14 days after pesticide application.

$$X=\frac{N1}{N}\times 100\%$$

$$Y=\frac{X1-X2}{X1}\times 100\%$$

where *X* is the disease incidence rate (%); N1 is the number of infected fruits; N is the total number of fruits investigated; *Y* is the control efficiency (%); X1 is the disease incidence rate of the control group; and X2 is the disease incidence rate of the treatment group.

### 2.9. Statistical Analysis

An ANOVA was performed by SPSS version 26.0 to test the significance. Data were compared with Duncan’s multiple range test and Tukey’s HSD test. Statistical significance was considered at *p* < 0.05.

## 3. Results

### 3.1. Identification of Strain F0b

**Figure 1:**
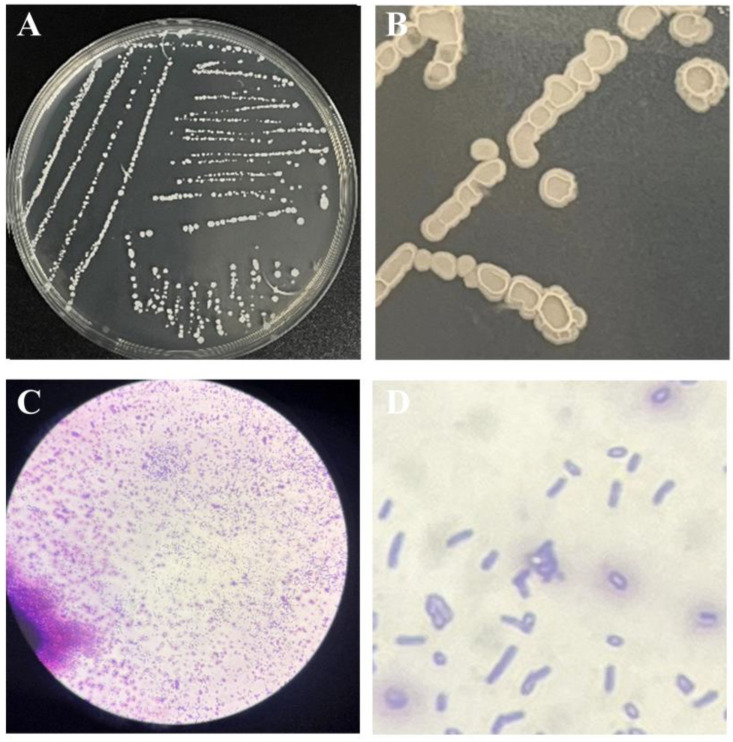
Morphological characteristics of strain F0b. (**A**,**B**) Colony morphology of strain F0b on NA medium. (**C**,**D**) Gram staining of strain F0b.

After 48 h of growth on NA medium, the morphology of the single F0b colony was light yellow, opaque, and emitted a distinct odor. In the early stages of cultivation, the colony surface was smooth, with neat round edges. In the later stages, the surface became wrinkled, the edges became irregular, and the colony spread in a cloud-like pattern (Figure 1A,B). The F0b strain was subjected to Gram staining and microscopic observation, which revealed a positive Gram reaction and rod-shaped bacteria (Figure 1C,D).

**Figure 2:**
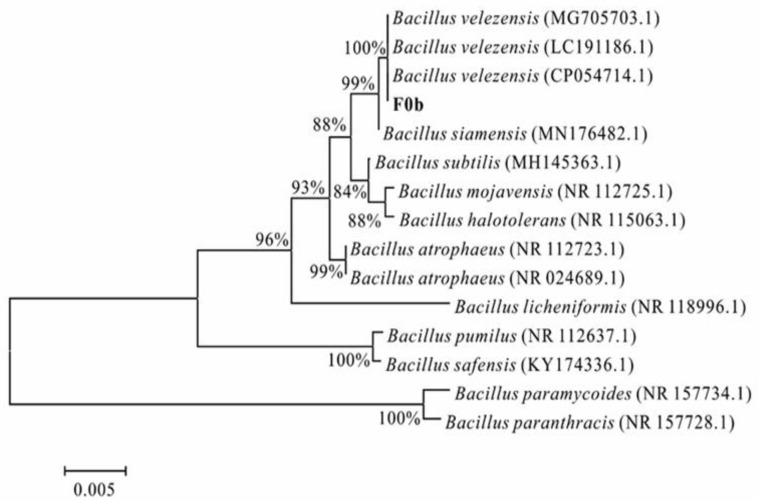
Phylogenetic analysis of 16S rDNA nucleotide sequence of F0b compared with those of *Bacillus* isolates.

As shown in the Figure 2, the 16S rDNA nucleotide sequence of F0b had high homologies to the corresponding sequences of *B. velezensis* MG705703.1 (100%), *B. velezensis* LC191186.1 (100%), and *B. velezensis* CP054714.1 (100%). Strain F0b was identified as *B. velezensis*.

### 3.2. Optimizing Fermentation Medium Compositions for Bacillus velezensis F0b

#### 3.2.1. Selection of Carbon, Nitrogen, and Mineral Sources by a Single Test

Based on the results of the above single-factor experiment, the addition amount of rice flour, ammonium chloride, and disodium phosphate exhibited a significant effect on the bacterial viability.

**Figure 3:**
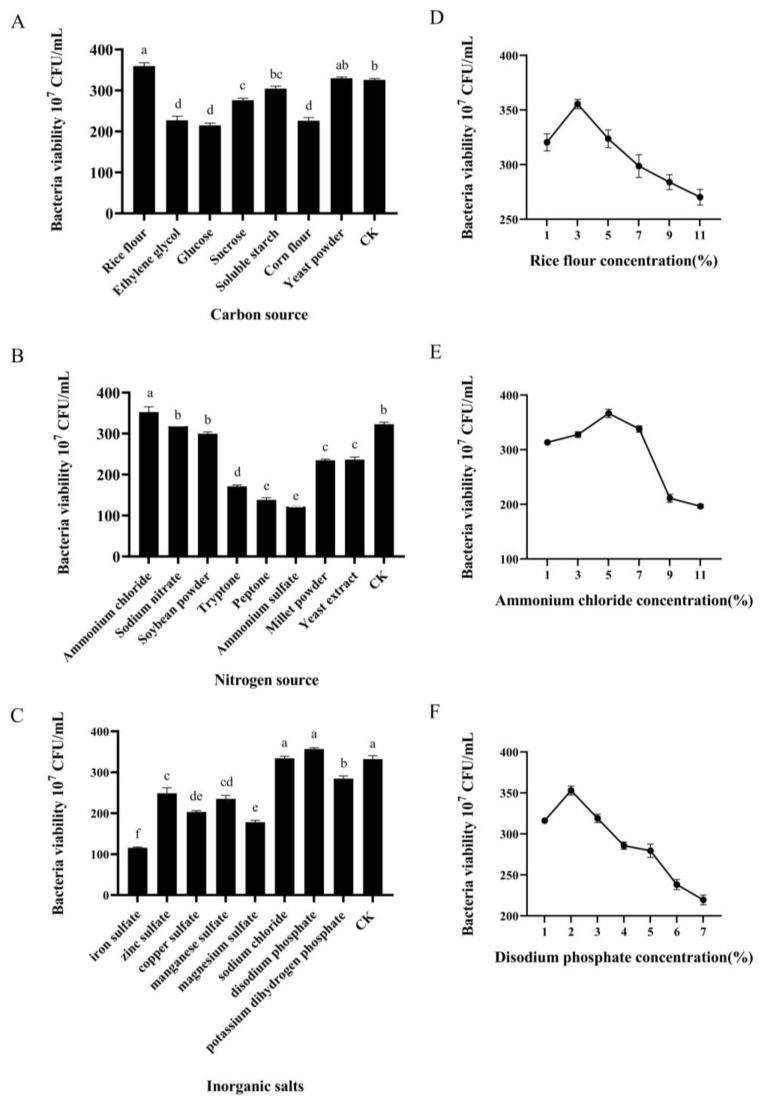
Effects of different carbon sources (**A**), nitrogen sources (**B**), and inorganic elements (**C**) on the fermentation of strain F0b. Effects of rice flour (**D**), ammonium chloride (**E**), and disodium phosphate (**F**) at different concentration levels. Data were compared with Tukey’s HSD test. Different lowercase letters mean significant difference (*p* < 0.05).

When rice flour was used as the carbon source, the number of viable bacteria in the fermentation medium reached its highest value at 3.60 × 10^9^ CFU/mL, which was significantly higher than that achieved with other carbon sources (Figure 3A,D). Consequently, rice flour was selected as the most suitable additional carbon source. The optimal bacterial viability, 3.56 × 10^9^ CFU/mL, was obtained when the rice flour concentration was 3%.

When ammonium chloride was added as the nitrogen source, the number of viable bacteria in the fermentation medium was the highest, reaching 3.53 × 10^9^ CFU/mL, which was significantly higher than that of other treatments (Figure 3B,E). Therefore, ammonium chloride was selected as the additional nitrogen source. As shown in Figure 3E, when the concentration of ammonium chloride was 5%, the number of viable bacteria reached 3.67 × 10^9^ CFU/mL. At concentrations either higher or lower than 5%, the number of viable bacteria decreases significantly. Therefore, a 5% concentration of ammonium chloride was selected as the optimal response value in the response surface.

When disodium phosphate was added as an inorganic salt, the number of live bacteria in the fermentation medium was the highest, reaching 3.57 × 10^9^ CFU/mL, which was significantly higher than the control group and other treatments. Therefore, disodium phosphate was selected as an additional inorganic salt. The result (Figure 3C,F) indicated that when the concentration of disodium phosphate was 2%, the number of viable bacteria reached 3.53 × 10^9^ CFU/mL, which was significantly higher than at other concentrations. Therefore, a disodium phosphate concentration of 2% was selected as the central value for the response surface analysis.

#### 3.2.2. Response Surface Methodology for Optimization of Medium Compositions

Using a response surface methodology based on the Box–Behnken design, a total of 17 combination experiments were conducted with various concentrations of rice flour, ammonium chloride, and disodium phosphate (Table 2). The results were analyzed to provide the following quadratic multinomial regression equation:

$$Y=417.75+5.09A-5.47B-2.56C+9.38AB+1.44AC-0.3125BC-6.02A^{2}-16.90B^{2}-14.46C^{2}$$

**Figure 4:**
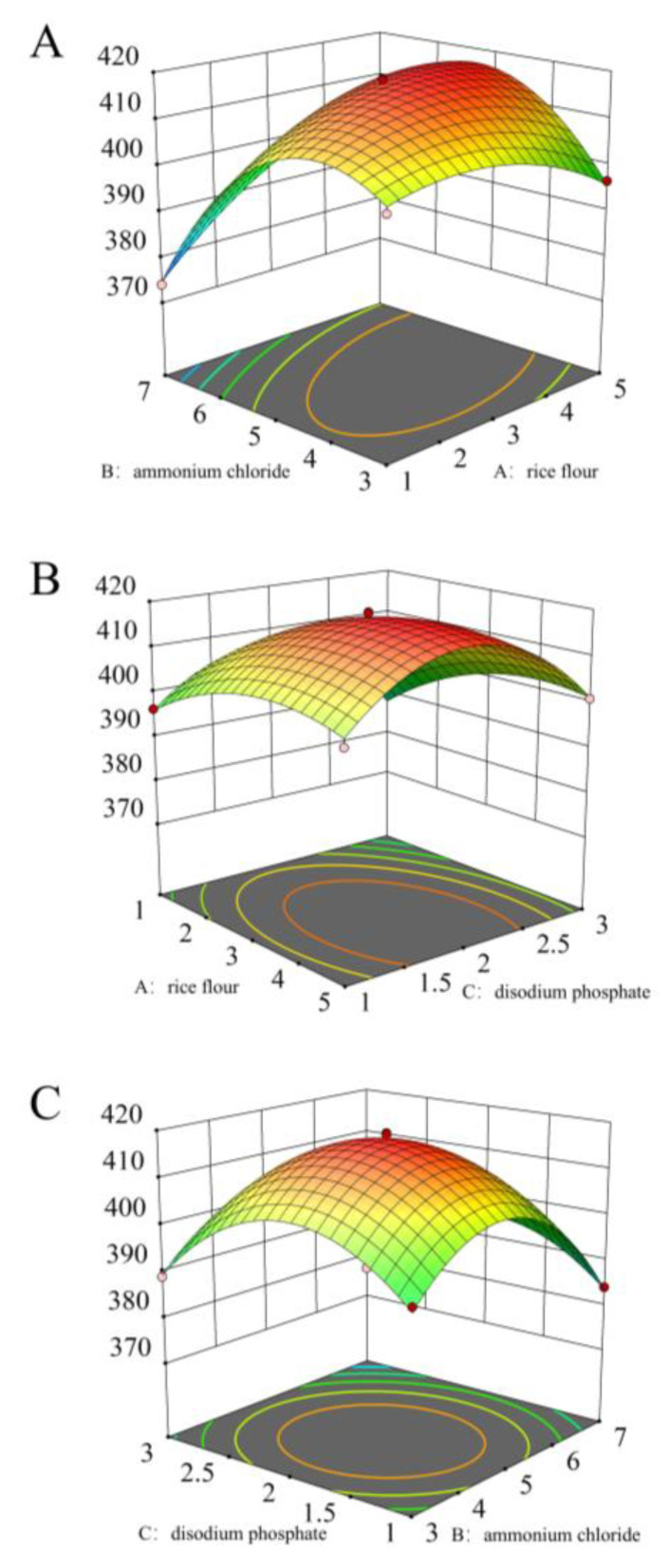
Response surface of the interaction among three factors of medium components, rice flour (**A**), ammonium chloride (**B**), and disodium phosphate (**C**) on the number of viable bacteria.

The response surface of the interaction among the three factors influencing the number of viable bacteria of *B. velezensis* F0b was illustrated (Figure 4). The interactions between the three factors—carbon source, nitrogen source, and inorganic salt—exhibit a parabolic relationship, with a maximum point observed. As the levels of these factors increase, the number of viable bacteria initially rises and then declines.

The optimized culture medium formula was adjusted to 3.472% rice flour, 4.898% ammonium chloride, and 1.871% disodium phosphate. Under these conditions, the average number of viable bacteria was 4.18 × 10^9^ CFU/mL, closely matching the predicted value of 4.19 × 10^9^ CFU/mL. Thus, this formulation was identified as the optimal fermentation medium source for *B. velezensis* F0b.

#### 3.2.3. Effects of Fermentation Conditions on *Bacillus velezensis* of Strain F0b

**Figure 5:**
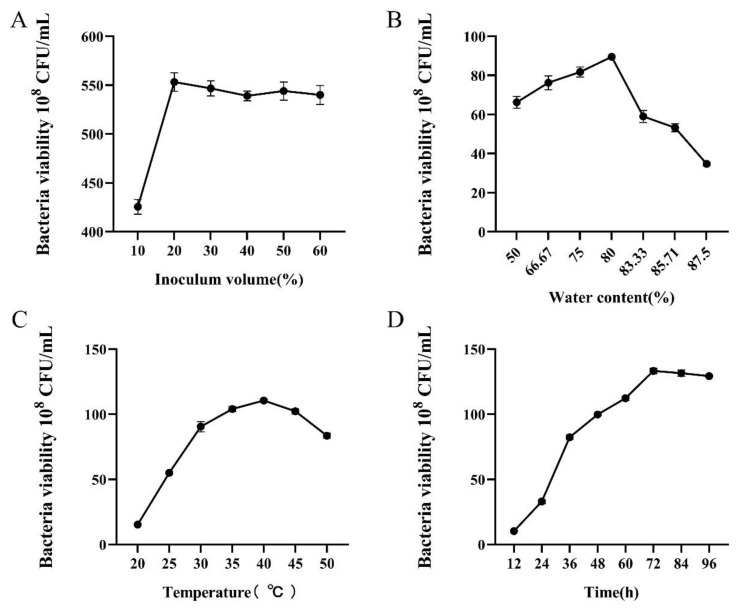
Effects of different inoculum (**A**), water content (**B**), temperature (**C**), and time (**D**) on the fermentation of strain F0b. Error bars represent standard error.

As shown in Figure 5A, when the inoculum volume was 20%, the number of viable bacteria was 5.53 × 10^9^ CFU/mL, which was highest. Increasing the inoculum volume over 20% did not make a significant difference in the number of viable bacteria of *B. velezensis* F0b, and a further increase showed no significant effect. Therefore, an inoculum volume of 20% was selected for the fermentation.

As shown in Figure 5B, the effect of varying water content in the culture medium on the viable bacterial count in the fermentation material of *B. velezensis* F0b was evaluated. The number of viable bacteria in strain F0b exhibited an initial increase followed by a decrease as the water content increased. When the water content reached 80%, the number of viable bacteria in the fermentation material reached 8.95 × 10^9^ CFU/mL. Both excessively high and low water content negatively impacted the number of viable bacteria. Therefore, the optimal water content for fermentation was determined to be 80%.

By analyzing the effects of different fermentation temperatures on the growth of *B. velezensis* F0b (Figure 5C), it was observed that the number of viable bacteria initially increased and then decreased with rising temperature. At 40 °C, the number of viable bacteria reached a maximum of 1.10 × 10^10^ CFU/mL, significantly exceeding the numbers observed at other temperatures. Temperatures either higher or lower than 40 °C inhibited the growth of strain F0b. Consequently, 40 °C was identified as the optimal fermentation temperature for *B. velezensis* F0b.

By evaluating the effects of different culture times on the fermentation of *B. velezensis* F0b (Figure 5D), it was observed that the number of viable bacteria steadily increased from 12 h to 72 h with significant differences. At 72 h, the number of viable bacteria reached 1.34 × 10^10^ CFU/mL. Beyond this point, no significant increase in the viable bacterial number was observed with extended fermentation time. Therefore, 72 h was determined to be the optimal fermentation time.

### 3.3. Screening of Carrier, Dispersant, and Wetting Agent

**Figure 6:**
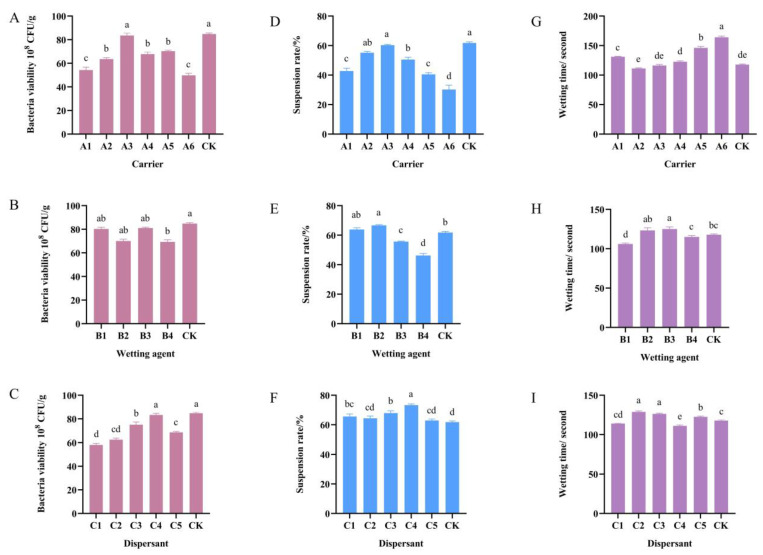
The effect of different carriers, wetting agents, and dispersants on the bacterial viability (**A**–**C**), suspension rate (**D**–**F**), and wetting time (**G**–**I**) of *Bacillus velezensis* F0b during 48 h of storage at 27 °C. CK are samples without any treatments stored at 27 °C. (**A**,**D**,**G**) Data were compared with Tukey’s HSD test. The remaining data were compared with Duncan’s multiple range test. Error bars represent standard error. Different lowercase letters mean a significant difference (*p* < 0.05).

The effect of different ingredients on the suspension rate, bacterial viability, and wetting time of *B. velezensis* F0b is illustrated in Figure 6.

Among the six carriers, only kaolinite (A3) did not differ significantly on the bacterial viability from that of the control (CK), with the number of viable bacteria remaining from 8.05 to 8.65 × 10^9^ CFU/g (Figure 6A). From the perspective of wetting time and the suspension rate, kaolinite exhibited the highest suspension rate when used as a carrier, significantly outperforming other materials (Figure 6D,G). It demonstrated good wettability, with a wetting time of 116.25 s, which while slightly longer than that of diatomite, showed no significant difference. Overall, when kaolinite is used as a carrier in the formulation of *B. velezensis* F0b wettable powder, it achieves the highest suspension rate, the best compatibility, and satisfactory wettability. Therefore, kaolinite is considered the most suitable carrier for preparing *B. velezensis* F0b wettable powder.

As wetting agents, B1 and B3 demonstrated good compatibility with *B. velezensis* F0b, with the number of viable bacteria exceeding 8 × 10^9^ CFU/g (Figure 6B). Upon the addition of B1, the suspension rate of the formulation was observed to be relatively high, reaching 63.86% (Figure 6E). Furthermore, the wetting time was 106 s, the shortest among all tested wetting agents, showing a significant difference compared to the others (Figure 6H). Therefore, B1 was selected as the wetting agent for subsequent experiments.

As dispersants, C1, C2, and C5 caused a significant reduction in the number of viable bacteria, indicating poor compatibility with the strain. In contrast, C3 and C4 had minimal impact on the viability of *B. velezensis* F0b, demonstrating good biocompatibility (Figure 6C). Specifically, the number of viable bacteria for sodium lignin sulfonate (C4) was 8.33 × 10^9^ CFU/g. Additionally, C4 exhibited the highest suspension rate at 73.33%, and its wetting time was relatively short at 111.00 s, significantly differing from other dispersants (Figure 6F,I). Consequently, C4 was selected as the dispersant for the wettable powder formulation of *B. velezensis* F0b.

### 3.4. Screening of the Ratio and Dosage of Wetting Agent and Dispersant

Based on the results of Section 3.3, the wetting agent sodium dodecyl sulfate (B1) and the dispersant sodium lignin sulfonate (C4) were selected. Subsequently, the effects of different ratios of B1 and C4 on the preparation of *B. velezensis* F0b were evaluated. The experimental results in Table 3 showed when the ratio of the wetting agent to dispersant was 3:7, the suspension rate reached 75.24%, and the wetting time was 99.75 s, showing a significant difference compared to other ratios tested. Therefore, sodium dodecyl sulfate (B1) and sodium lignin sulfonate (C4) were employed as compound wetting agents in a ratio of 3:7.

As shown in Table 4, when the compound is added at a concentration of 16%, the suspension rate reaches 80.92% with a wetting time of 89.25 s, indicating both excellent suspension and wettability. Therefore, the additional amount of the wetting agent in the formulation is 16%.

### 3.5. Screening of UV Protectants

**Figure 7:**
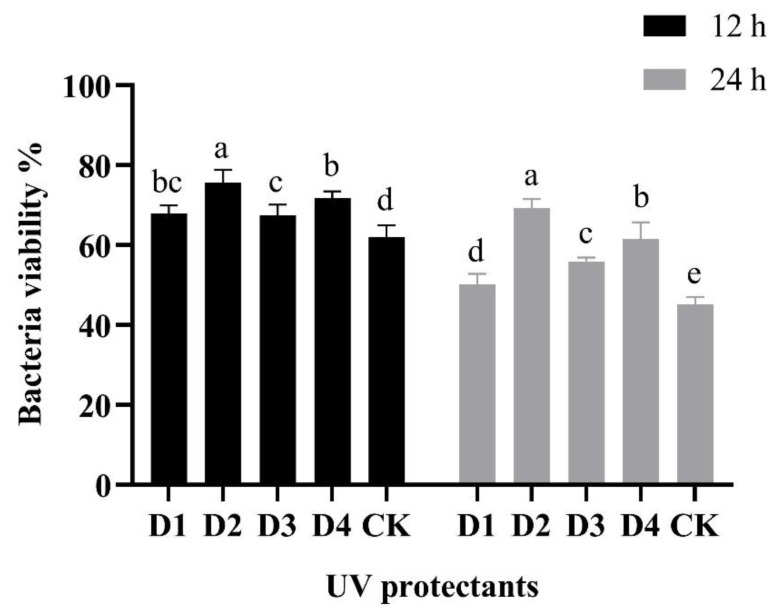
Effect of different UV protectants on the bacterial viability of *Bacillus velezensis* F0b after 12 h and 48 h of storage at 27 °C. CK are samples without any treatments stored at 27 °C. Data were compared with Duncan’s multiple range test. Error bars represent standard deviations. Different lowercase letters mean significant difference (*p* < 0.05).

Different UV protectants were mixed uniformly with the fermentation broth. The results indicated that when ascorbic acid was utilized as the UV protectant, the survival rates after 12 h and 24 h of UV irradiation were 75.62% and 69.27%, respectively (Figure 7). The survival rate of *B. velezensis* was significantly higher than that of soluble starch, xanthan gum, and sodium alginate. Therefore, ascorbic acid was selected as the UV protectant for *B. velezensis* F0b wettable powder. As the dosage of ascorbic acid increased from 1% to 2%, the survival rate of *B. velezensis* F0b demonstrated a significant improvement. At a dosage of 2%, the survival rates after 12 h and 24 h were 77.24% and 68.20%, respectively, both significantly higher than those observed at the 1% dosage. However, when the dosage exceeded 2%, the differences were not statistically significant. Therefore, the optimal dosage of ascorbic acid was determined to be 2%.

### 3.6. Physical Properties of Wettable Powder of F0b

The *B. velezensis* F0b wettable powder was successfully developed. As indicated by the test results in Table 5, the formulation exhibits favorable physical properties and complies with required standards.

### 3.7. Field Experiments

The field efficacy test results indicated that 7 days after the second application, *B. velezensis* F0b wettable powder (WP) achieved a control efficacy of 50.58–64.20% against strawberry gray mold caused by *Botrytis cinerea* at dosages of 100 g/667 m^2^, 200 g/667 m^2^, and 300 g/667 m^2^ compared to a control efficacy of 57.20% with the *Trichoderma* WP control agent (Table 6). Fourteen days after the second application, the control efficacy of F0b WP increased to 58.29–73.14% at the same dosages, while the control efficacy of *Trichoderma* WP was 63.71%. Notably, at a dosage of 300 g/667 m^2^, the F0b WP preparation showed a significantly higher control effect on *Botrytis cinerea* than the *Trichoderma* WP preparation at 200 g/667 m^2^ during the same period.

## 4. Discussion

Microbial pesticides are effective in preventing and controlling plant diseases while also promoting crop growth, thus contributing to the reduction in chemical pesticide usage. The development of microbial pesticides is a key direction for advancing environmentally friendly plant disease management [23,24]. *Bacillus* species, being the most widely studied biocontrol bacteria in microbial pesticide research and applications [25,26,27], play a crucial role in this field. *B. velezensis*, as a novel biocontrol agent, holds significant potential for the development and application of microbial pesticides [28,29]. Its use is of considerable importance for the green and sustainable development of agriculture.

However, in practical applications, *B. velezensis* is susceptible to various factors, such as host conditions, climate, soil, and water quality. These factors can interfere with the growth and reproduction of the strain, thereby affecting its biological control efficacy. Consequently, there is an urgent need to isolate additional strains with strong environmental adaptability, optimize fermentation conditions, and develop superior formulations. This approach is of great significance for enhancing the biological control of plant diseases.

Shi et al. [30] optimized the fermentation medium and growth conditions for *B. velezensis* BHZ-29 and found that the best medium formulation was achieved when molasses, peptone, and magnesium sulfate were used as the carbon source, nitrogen source, and inorganic salt, respectively. Ma Ting et al. [31] optimized the fermentation of *Bacillus amyloliquefaciens* 3–5 and determined the optimal medium formula including maize starch, beef extract, and MgSO_4_. The carbon source, nitrogen source, and inorganic salt materials used in the fermentation medium are similar to those employed for *B. velezensis* F0b in this study. Furthermore, optimizing fermentation substrates not only promotes bacterial growth but may also enhance the antimicrobial activity of *B. velezensis*. A key antimicrobial mechanism of *B. velezensis* involves the production of bioactive compounds, such as antimicrobial peptides and enzymes, which inhibit the growth of pathogenic microorganisms. Previous studies have demonstrated that nitrogen plays a crucial role in the synthesis of both biomass and secondary metabolites essential for microbial growth and metabolism, with ammonia being the preferred nitrogen source [32,33]. In the present study, ammonium chloride was chosen as the nitrogen source for the fermentation medium, and it is hypothesized that this choice may have facilitated the production of antimicrobial metabolites in *B. velezensis*.

Ma Ting et al. [31] found that the best fermentation conditions include an inoculum volume of 6%, a ratio of material to sterile water of 1:1, and a fermentation time of 44 h. Xu et al. [34] optimized the fermentation conditions of *Bacillus atrophaeus* XHG-1-3m2 and found that a 3% inoculum volume, fermentation time of 24 h, and 40% liquid volume were the best. In contrast, the fermentation process of *B. velezensis* F0b in this study required a larger inoculum amount and a longer fermentation time, as well as a higher content of sterile water. The higher inoculum volume and longer fermentation time could have contributed to the efficient colonization of the fermentation medium, providing optimal conditions for both growth and the production of antimicrobial compounds. Additionally, the higher content of sterile water may have supported better bacterial dispersion and nutrient availability during the fermentation process, enhancing bacterial survival and growth.

The optimization strategy of adjuvants is crucial to ensure the stability and effectiveness of biocontrol agents under different environmental conditions. By screening carriers, additives, stabilizers, and UV protectants, Zhuangyuan et al. [35] developed a wettable powder formulation of *Bacillus subtilis* 262XY2x and finally determined that sodium dodecyl sulfate was used as an auxiliary agent and ascorbic acid was used as a UV protectant, which aligns with the findings of this study. Similarly, Shen et al. [36] determined the formulation of *B. velezensis* SH-1471 WP, using kaolinite as a carrier, sodium lignin sulfonate as a wetting agent, NNO as a dispersant, and ascorbic acid as a UV protectant. The carrier, wetting agent, and ascorbic acid selected in this experiment are consistent.

Russi et al. [37] determined the effectiveness of *B. velezensis* S26 endospore-rich suspension in managing gray mold and anthracnose in strawberries during the postharvest phase. Our study further supports these findings, as the F0b WP formulation exhibited strong efficacy in controlling strawberry gray mold caused by *Botrytis cinerea*. The superior performance of F0b WP can be attributed to the optimized fermentation conditions, which enhanced the strain’s antimicrobial activity, and the well-chosen formulation materials, which ensured its stability and efficacy during application.

In conclusion, this study successfully optimized the fermentation conditions for *B. velezensis* F0b, developed a stable wettable powder formulation, and demonstrated its promising potential as a microbial pesticide for controlling strawberry gray mold caused by *Botrytis cinerea*. Our findings demonstrate that F0b WP is a promising microbial fungicide, with strong potential for controlling *Botrytis cinerea* and possibly other plant diseases. Further studies are needed to explore the detailed mechanisms underlying its antimicrobial activity and to assess its effectiveness against other plant pathogens, which enables its wider application in sustainable agriculture.

## Figures and Tables

**Table 1:** Factor and level of Box–Behnken design.

Code	Variables	Level		
		−1	0	1
A	Rice flour concentration (%)	1	3	5
B	Ammonium chloride concentration (%)	3	5	7
C	Disodium phosphate concentration (%)	1	2	3

**Table 2:** The results of Box–Behnken optimization experiment.

Run Numbers	A	B	C	The Number of Viable Bacteria (1 × 10^9^ CFU/mL)
1	−1	1	0	3.74
2	0	0	0	4.16
3	0	0	0	4.19
4	0	1	−1	3.84
5	0	1	1	3.77
6	−1	0	1	3.90
7	−1	0	−1	3.96
8	1	1	0	4.05
9	0	0	0	4.18
10	0	−1	−1	3.95
11	1	0	1	4.01
12	0	−1	1	3.89
13	1	−1	0	3.97
14	−1	−1	0	4.03
15	1	0	−1	4.02
16	0	0	0	4.17
17	0	0	0	4.18

**Table 3:** Effects of different ratios of wetting agent to dispersant on *Bacillus velezensis* F0b WP formulation.

Wetting-Agent-to-Dispersant Ratio	Suspension Rate (%)	Wetting Time (s)
1:9	70.54 ± 1.03 bc	120.00 ± 1.47 a
2:8	72.07 ± 0.70 b	112.00 ± 0.71 bc
3:7	75.24 ± 1.46 a	99.75 ± 1.44 e
4:6	68.35 ± 0.76 c	115.25 ± 1.32 ab
5:5	69.85 ± 0.79 bc	108.75 ± 1.18 cd
6:4	68.17 ± 0.90 c	116.00 ± 2.12 ab
7:3	63.95 ± 0.88 d	117.25 ± 2.93 ab
8:2	68.07 ± 0.55 c	115.50 ± 1.44 ab
9:1	64.91 ± 0.79 d	104.75 ± 1.65 d

Different lowercase letters mean significant difference (*p*  <  0.05).

**Table 4:** Effects of different concentrations of wetting agent on *Bacillus velezensis* F0b WP formulation.

Wetting Agent (%)	Suspension Rate (%)	Wetting Time (s)
2	64.99 ± 1.44 g	115.00 ± 1.41 a
4	64.87 ± 0.64 g	109.00 ± 1.63 b
6	67.71 ± 0.80 f	109.75 ± 1.38 b
8	70.90 ± 0.93 e	106.00 ± 1.47 b
10	73.97 ± 0.93 d	100.75 ± 1.49 c
12	75.08 ± 0.70 cd	97.00 ± 0.71 cd
14	76.91 ± 0.37 bc	95.25 ± 0.95 d
16	80.92 ± 0.41 a	89.25 ± 0.85 e
18	78.33 ± 0.22 b	85.50 ± 1.19 e
20	78.47 ± 0.72 b	85.75 ± 1.89 e

Different lowercase letters mean significant difference (*p*  <  0.05).

**Table 5:** Physical properties of wettable powder of F0b.

Properties	Description
Spore viability (10^8^ CFU g^−1^)	70
pH	6.44
Wetting time (s)	86.33
Suspension rate (%)	78.55

**Table 6:** Field control effect of F0b WP on strawberry gray mold caused by *Botrytis cinerea*.

Treatment	7 Days After Spraying		14 Days After Spraying	
	Disease Incidence (%)	Control Efficiency (%)	Disease Incidence (%)	Control Efficiency (%)
1	12.70 ± 0.57 b	50.58 b	14.60 b	58.29 c
2	11.40 ± 0.26 bc	55.64 ab	12.00 b	65.14 b
3	9.20 ± 0.71 c	64.20 a	9.40 c	73.14 a
4	11.00 ± 1.67 bc	57.20 ab	12.70 b	63.71 bc
5	25.70 ± 0.38 a	-	35.00 a	-

Different lowercase letters mean significant difference (*p* < 0.05).

## Data Availability

The original contributions presented in this study are included in the article. Further inquiries can be directed to the corresponding authors.
